# *Crataegus ×ninae-celottiae* and *C. ×cogswellii* (Rosaceae, Maleae), two spontaneously formed intersectional nothospecies

**DOI:** 10.3897/phytokeys.36.6784

**Published:** 2014-04-09

**Authors:** Knud Ib Christensen, Mehdi Zarrei, Maria Kuzmina, Nadia Talent, Charlotte Lin, Timothy A. Dickinson

**Affiliations:** 1 Assoc. Prof. M.Sc. Ph.D. Knud Ib Christensen (born 13 October 1955, deceased 16 January 2012), formerly at the Botanical Garden, Natural History Museum of Denmark, University of Copenhagen; 2 The Centre for Applied Genomics (TCAG), The Hospital for Sick Children, Peter Gilgan Centre for Research and Learning, Rm. 139715, 686 Bay St., Toronto, Ontario, M5G 0A4; 3 Department of Botany, MRC-166 National Museum of Natural History Smithsonian Institution Rm W106 Washington, DC 20013-7012 USA; 4 Green Plant Herbarium (TRT), Department of Natural History, Royal Ontario Museum, 100 Queen’s Park, Toronto, Ontario Canada M5S 2C6; 5 School of Education, University of Stirling, Scotland UK FK9 4LA; 6 Department of Ecology & Evolutionary Biology, University of Toronto, 25 Willcocks St., Toronto, Ontario, Canada M5S 3B2

**Keywords:** North America, hawthorn, hybridization, diploid, leaf shape, ITS2, DNA barcodes

## Abstract

*Crataegus monogyna* Jacq. is naturalized in North America, where it has hybridized with native diploid hawthorns at least twice. We provide names for the two nothospecies (as well as for the corresponding nothosections and nothoseries), referring to existing documentation in the literature for **nothosp. nov.** *Crataegus* ×*ninae-celottiae* K.I. Chr. & T.A. Dickinson (*C. monogyna* × *C. punctata* Jacq.). New data are provided to further document **nothosp. nov.** *Crataegus* ×*cogswellii* K.I. Chr. & T.A. Dickinson (*C. monogyna* × *C. suksdorfii* (Sarg.) Kruschke). In both cases, the striking differences in leaf shape between most New World hawthorns and Old World section *Crataegus*, and the intermediacy of the hybrids, account for the relative ease with which these hybrids can be recognized. Finally, new sequence data from ITS2 and chloroplast DNA barcoding loci confirm the genetic relationships between the two nothospecies and their respective parents.

## Introduction

*Crataegus monogyna* Jacq. is a widespread species of *Crataegus* sect. *Crataegus* that occurs in much of Europe, northern Africa and western Asia. Within the area of its natural distribution it hybridizes with several other species of sect. *Crataegus*, e.g., *Crataegus laevigata* (Poir.) DC., *Crataegus rhipidophylla* Gand., *Crataegus meyeri* Pojark., *Crataegus pentagyna* Waldst. & Kit. ex Willd., *Crataegus orientalis* M. Bieb., and *Crataegus azarolus* L., as well as *Crataegus nigra* Waldst. & Kit. of sect. *Sanguineae* (Albarouki and Peterson 2007; Byatt 1975; Christensen 1983; Christensen 1992a, b, 1994; Christensen and Zielinski 2008; Dönmez 2004). In fact, Christensen (1992) applied the term “compilospecies” to *Crataegus monogyna*. This term, coined by Harlan and DeWet (1963), describes species that aggressively acquire genes from other species by introgressive hybridization, potentially explaining the “…great variability of *Crataegus monogyna* and also its wide distribution” in the Old World (Christensen 1992a). *Crataegus monogyna* was introduced to the U.S.A. and Canada by the early European settlers (Billings 1862; Douglas 1914; Kirk 1819; Provancher 1863). It has often escaped from cultivation and, e.g., in abandoned fields and woodlands with extensive hawthorn colonization, it may hybridize with native diploid species of *Crataegus* such as *Crataegus punctata* Jacq. (sect. *Coccineae* Loudon; Phipps pers. comm.; Wells and Phipps 1989) and *Crataegus suksdorfii* (Sarg.) Kruschke (sect. *Douglasia* Loudon; Dickinson et al. 2008; Love and Feigen 1978; Talent and Dickinson 2005). Because of the striking contrast in leaf shape between members of *Crataegus* sect. *Crataegus* and most North American *Crataegus* species, these hybrids are currently the best-known examples of diploid-diploid hybridization in the North American *Crataegus* flora. We provide names for these two nothospecies (as well as for the corresponding nothosections and nothoseries), referring to existing documentation in the literature for *Crataegus ×ninae-celottiae* K.I. Chr. & T.A. Dickinson (*Crataegus monogyna* × *Crataegus punctata* Jacq.; Wells and Phipps 1989). We also document variation in leaf shape for the second hybrid, *Crataegus ×cogswellii* K.I. Chr. & T.A. Dickinson (*Crataegus suksdorfii* × *Crataegus monogyna*), and provide new sequence data from ITS2 and chloroplast DNA barcoding loci that confirm the genetic relationships between the two nothospecies and their respective parents.

## Methods

**Sampling.** Because the occurrence of *Crataegus monogyna* and its hybrids is sporadic, most of our samples are non-random, and merely attempt to document the co-occurrence of the parental species and (or) their hybrids (Table 1). Only in the case of the hybrid swarm found at the Cogswell-Foster Preserve in Linn Co., Oregon (site OR1), have we used either the throw of a pair of dice or ignorant person sampling (Ward 1974) in order to draw more unbiased samples, with the inevitable consequence that these samples reflect the greater frequency of the introduced species and its hybrids. To mitigate this, we have included individuals of mostly diploid *Crataegus suksdorfii* from other sites in order to reflect the variation found in this taxon.

**Table 1.** Sites in Canada and the United States at which collections of native and naturalized diploid (unless indicated otherwise) *Crataegus* were made as vouchers for morphological, chemical, and molecular (boldface) observations (Fig. 1–3; Tables 2–4). Sampled individuals are listed by their collector and collection number; principal collector is T. A. Dickinson (D) unless indicated otherwise, as follows: JC, J. Coughlan; CAR, Rebecca Dotterer; EH, E. Harris; EL, E. Y. Y. Lo; RML, R. M. Love; MP, M. A. Purich; Z, P. Zika.

State/Province				
	Site	Location	Taxon	Individuals
British Columbia				
	BC16	Central Kootenay R.D., Robson, Broadwater Road, Broadwater Road S side	*Crataegus monogyna*	**2008-26**
	BC	Central Kootenay R.D., Winlaw, next to Winlaw general store (10 miles S of Slocan) on bank of small creek (tributary of Slocan River).	*Crataegus suksdorfii* Probably polyploid	RML9313
California				
	CA11	Humboldt Co., Hwy 36, 6.8 air km W of Bridgeville	*Crataegus monogyna*	**JC001**
	CAR4	Trinity Co., T37N R7W S17	*Crataegus suksdorfii* Polyploid?	CAR042
	CAR5	Siskiyou Co., flood plain of the Scott R., N side of Fay Lane, between jct. Hwy 3 and bridge	*Crataegus suksdorfii*	**2006-16, 2006-18, 2006-19, 2006-22**, CAR044
	CAR7	Siskiyou Co., T26N R11W S17	*Crataegus suksdorfii* Polyploid?	CAR048
	CRRR01	Sonoma Co., Ragle Ranch, W of Sebastopol	*Crataegus monogyna*	**JC003**
Idaho				
	ID10	Benewah Co., T44N R1W S8, Soldier Creek, W side of Hwy 3 just N of RR crossing and St. Mary’s R.	*Crataegus suksdorfii* Probably polyploid	D1608
Montana				
	MT1	Powell Co., Dry Creek, N side, edge of meadow and gallery forest	4× *Crataegus suksdorfii*	D1614, D1619
Ontario				
	NTON23	City of Toronto, Centennial Park, Etobicoke	*Crataegus punctata*	**MP71**
			*Crataegus ×ninae-celottiae*	**MP24, MP73**
	ON21	Bruce Co., Eastnor Twp., Barrow Bay, E side Hwy 9 at S.R. 15	*Crataegus punctata*	Dickinson & Nguyen **BB4**
	ON31	Middlesex Co., Ilderton, SE corner Denfield Side Road and Ilderton Road (Hwy 16)	*Crataegus punctata*	EH52, **MP56, MP61, 2003-79**
	ON40	City of Toronto, Ashbridges Bay Park	*Crataegus punctata*	**MP35**
	ON45	Durham R.M., Bowmanville, floodplain of Bowmanville Creek	*Crataegus monogyna*	**MP82, MP83, MP98**
			*Crataegus ×ninae-celottiae*	2002-13, **MP84, MP85, MP86**
			*Crataegus punctata*	**MP81**
	ON46	Perth Co., E side Thames R. North Branch 2 km S of Motherwell	*Crataegus punctata*	**2008-72A**
Oregon				
	OR1	Linn Co., Willamette Valley, Cogswell-Foster Preserve	*Crataegus monogyna* (diploid)	**EL74**, EL78, EL80, EL83, OR1-5, OR1-8, OR1-9, OR1-10, OR1-11, OR1-12, OR1-16
			Triploid *Crataegus monogyna*	**RML C-2003-25**
			*Crataegus ×cogswellii*	99FW7-1, 99FW7-2, 99FW7-3, 99FW7-6, 99FW7-7, 99FW7-8, 99FW7-9, 2009-36, EL68, **EL71**, EL73, EL76, EL77, **EL79**, EL81, EL82, EL84, **EL85**, OR1-2, OR1-3, OR1-4, OR1-6, OR1-7, OR1-13, OR1-14, OR1-15, OR1-17, OR1-18, OR1-19, OR1-20, RML8718
			*Crataegus suksdorfii*	**EL68**, EL69, EL72, EL75, OR1-1, RML8709
	OR	Lane Co, City of Eugene	*Crataegus ×cogswellii*	RML C-2003-12, RML C-2003-13, RML9304
	OR4	Douglas Co., Upper Elk Meadow, 28 miles SSE Cottage Grove	*Crataegus suksdorfii* Probably polyploid	RML8758, RML8767, RML8768
	OR11	Columbia Co., Sauvie Island, Willow Park Island, Willow Bar Islands beach, just N of Columbia-Multnomah county line, on bank of Columbia River	*Crataegus monogyna*	**EL108**
			*Crataegus ×cogswellii*	Z18482
			*Crataegus suksdorfii*	**JC117, JC118, JC119**
	OR18	Jackson Co., Rogue River, Old Stage Rd. 80 m NE of Rogue River Hwy/99	*Crataegus suksdorfii*	**JC039**
	OR22	Linn Co., Corvallis, KOA Campground, 440 m from hwy 34 on Oakville Rd. SW. specimen 150 m SE of camp entrance	*Crataegus suksdorfii*	**JC060**
	OR35	Skamania Co., Cascade Locks, 110 m N of Cascade Locks Rd., on N side of Forest Ln.	*Crataegus suksdorfii*	**JC092**
	OR37	Multnomah Co., Columbia River Gorge National Scenic Area, 1.5 km NE of Troutdale	*Crataegus suksdorfii*	**JC098**, **JC102**
	OR38	Columbia Co., Diblee Pt., Site 350 m N of Dike Rd., 1.8 km WNW of Lewis and Clark Bridge	*Crataegus suksdorfii*	**JC136**
Washington				
	WA	Clark Co. S of mouth of Lewis River, ca. 1.5 air miles NNW of Ridgefield	*Crataegus suksdorfii*	**Z18485**
	WA8	Skamania Co., Gifford Pinchot National Forest, Zig Zag Lake, 9 mi NW of Wind R.	*Crataegus suksdorfii* Probably polyploid	Brooks s.n.
	WA10	Skamania Co., Gifford Pinchot National Forest, Upper Goose Creek Meadow	*Crataegus suksdorfii* Probably polyploid	RML8909

**Table 2.** Results of Neighbor-joining clustering of sequence data for chloroplast DNA barcode loci. GenBank accession numbers indicate cluster affiliation (Cluster 1 or 2) for *Crataegus* species and their putative hybrids. Details of the BOLD data can be found at dx.doi.org/10.5883/DS-CRATMONO. See Table 1 for sites and collectors; eight-digit ROM Green Plant Herbarium (TRT) accession numbers identify vouchers.

Taxon / site / BOLD / tree / TRT	Cluster 1 – sections *Coccineae* and *Douglasia*		Cluster 2 – section *Crataegus*	
	*rbcL-a*	*trnH-psbA*	*rbcL-a*	*trnH-psbA*
*Crataegus punctata*				
NTON23 TRT103 MP71 TRT00002237	KC251377	KC251652		
ON31 TRT096 MP61 TRT00002228	KC251375	KC251650		
ON31 TRT105 MP56 TRT00002223	KC251372	KC251647		
ON40 TRT101 MP35 TRT00002203	KC251374	KC251649		
ON45 TRT104 MP81 TRT000047	KC251378	KC251653		
ON46 TRT210 2008-72A TRT00000908	KC251373	KC251648		
*Crataegus ×ninae-celottiae*				
NTON23 TRT106 MP24 TRT00002199			KC251376	KC251651
NTON23 TRT203 MP73 TRT00002239	KC251350	KC251624		
ON45 TRT201 MP85 TRT00002250			KC251348	KC251622
ON45 TRT202 MP86 TRT00002251			KC251351	KC251625
ON45 TRT204 MP84 TRT00002249			KC251349	KC251623
*Crataegus monogyna*				
BC16 TRT209 2008-26 TRT00002452			KC251343	KC251617
CA11 TRT274 JC001 TRT00020101			KC251338	KC251612
CRRR01 TRT275 JC003 TRT00020102			KC251341	KC251615
ON31 TRT109 2003-79 TRT00000395			KC251340	KC251614
ON45 TRT108 MP82			KC251342	KC251616
ON45 TRT190 MP83 TRT00002248			KC251339	KC251613
ON45 TRT211 MP98 TRT00029476			KC251336	KC251610
OR1 TRT005 EL80 TRT00000413			KC251347	KC251621
OR1 TRT006 EL83 TRT00000415			KC251346	KC251620
OR1 TRT007 EL74 TRT00000416			KC251344	KC251618
OR TRT030 RML C-2003-25 TRT00000420			KC251337	KC251611
OR11 TRT143 EL108 TRT00000417			KC251345	KC251619
*Crataegus ×cogswellii*				
OR1 TRT206 EL71 TRT00002650		KC251627		
OR1 TRT207 EL85 TRT00002654		KC251626		
OR1 TRT208 EL79 TRT00002657	KC251352			
*Crataegus suksdorfii*				
CAR5 TRT129 2006-19 TRT00001569	KC251419	KC251692		
CAR5 TRT133 2006-22 TRT00001563	KC251418	KC251691		
CAR5 TRT140 2006-16 TRT00001567	KC251417	KC251690		
CAR5 TRT141 2006-18 TRT00001568	KC251416	KC251689		
OR1 TRT205 EL68 TRT00001724	KC251424	KC251699		
WA TRT146 Z18485 TRT00001805	KC251415	KC251688		

**Table 3.** Voucher specimens for cloned ITS2 data, listing site number (Table 1), collection number, ROM Green Plant Herbarium (TRT) accession numbers, and the GenBank accession numbers for individual clones.

Taxa	Voucher	GenBank accession number
*Crataegus suksdorfii*	OR18 *Coughlan, Zarrei, and Shaw* JC039 (TRT00020137)	KC173887, KC173888, KC173889, KC173890, KC173891, KC173892, KC173893
	OR22 *Coughlan, Zarrei, and Shaw* JC60 (TRT00020146)	KC173587, KC173588, KC173589, KC173590, KC173591, KC173592
	OR35 *Coughlan, Zarrei, and Shaw* JC092 (TRT00020153)	KC173957, KC173958, KC173959, KC173960, KC173961, KC173962, KC173963, KC173964
	OR37 *Coughlan, Zarrei, and Shaw* JC98 (TRT00020159)	KC173595, KC173596, KC173597, KC173598, KC173599, KC173600, KC173601, KC173602, KC173603, KC173604
	OR37 *Coughlan, Zarrei, and Shaw* JC102 (TRT00020163)	KC174113, KC174114, KC174115, KC174116, KC174117
	OR11 *Coughlan, Zarrei, and Shaw* JC117 (TRT00020172)	KC174118, KC174119
	OR11 *Coughlan, Zarrei, and Shaw* JC118 (TRT00020232)	KC174178, KC174179, KC174180, KC174181, KC174182, KC174183
	OR11 *Coughlan, Zarrei, and Shaw* JC119 (TRT00020234)	KC174144, KC174145, KC174146, KC174147, KC174148, KC174149, KC174150
	OR38 *Coughlan, Zarrei, and Shaw* JC136 (TRT00020242)	KC173605, KC173606, KC173607, KC173608, KC173609
	CAR5 *Dickinson and Lo* 2006-16 (TRT00001567)	KC173531, KC173532, KC173533, KC173534, KC173535, KC173536, KC173537, KC173538
	CAR5 *Lo and Dickinson* 2006-22 (TRT00001563)	KC173522, KC173523, KC173524, KC173525, KC173526, KC173527, KC173528, KC173529, KC173530
	OR1 *Lo, Dickinson, and Nguyen* EL-68 (TRT00001724)	KC173577, KC173578, KC173579, KC173580, KC173581, KC173582, KC173583, KC173584, KC173585, KC173586
	WA *Zika* 18485 (=18430, 18417; TRT00001805)	KC173513, KC173514, KC173515, KC173516, KC173517, KC173518, KC173519, KC173520, KC173521
*Crataegus ×cogswellii*	OR1 *Lo, Dickinson, and Nguyen* EL-71 (TRT00002650)	KC173663, KC173664, KC173665, KC173666, KC173667, KC173668
	OR1 *Lo, Dickinson, and Nguyen* EL-79 (TRT00002657)	KC173682, KC173683, KC173684, KC173685, KC173686, KC173687
	OR1 *Lo, Dickinson, and Nguyen* EL-85 (TRT00002654)	KC173669, KC173670, KC173671, KC173672, KC173673, KC173674, KC173675, KC173676, KC173677, KC173678, KC173679, KC173680, KC173681
*Crataegus monogyna*	OR1 *Lo, Dickinson, and Nguyen* EL-74 (TRT00000416)	KC173650, KC173651, KC173652, KC173653, KC173654
	BC16 *Dickinson, Lee, and Talent* 2008-26 (TRT00002452)	KC173655, KC173656, KC173657, KC173658, KC173659, KC173660, KC173661, KC173662
	ON45 *Purich* MP98 (TRT00029476)	KC173643, KC173644, KC173645, KC173646, KC173647, KC173648, KC173649
*Crataegus ×ninae-celottiae*	ON45 *Purich and Talent* MP84 (TRT00002249)	KC174184, KC174185, KC174186, KC174187, KC174188, KC174189
	ON45 *Purich and Talent* MP85 (TRT00002250)	KC174190, KC174191, KC174192, KC174193, KC174194, KC174195
	ON45 *Purich and Talent* MP86 (TRT00002251)	KC173688, KC173689, KC173690, KC173691, KC173692, KC173693
*Crataegus punctata*	ON21 *Dickinson and Nguyen BB4* (TRT)	KC174266, KC174267, KC174268, KC174269, KC174270, KC174271
	ON31 *Purich* s.n (TRT)	KC174272, KC174273, KC174274, KC174275
	NTON23 *Purich, Talent, Nguyen*, *and Lo MP73* (TRT00002239)	KC173694, KC173695, KC173696, KC173697, KC173698, KC173699, KC173700, KC173701

Note that we distinguish the taxon referred to here as *Crataegus suksdorfii* from the other western North American black-fruited hawthorn with 20 stamens per flower, *Crataegus gaylussacia* A. Heller. This is because these two taxa are allopatric (Coughlan 2012 and unpubl. data), and differ in morphology and cytotype. *Crataegus gaylussacia* has shorter petioles and thorns that are thicker at their base than is the case with diploid *Crataegus suksdorfii* (Dickinson unpubl. data). Molecular data are consistent with *Crataegus gaylussacia* being an autotriploid derivative of diploid *Crataegus suksdorfii* (Zarrei et al. http://2012.botanyconference.org/engine/search/index.php?func=detail&aid=536 and unpubl. data; see also Lo et al. 2009). In contrast, the *Crataegus suksdorfii* complex has been shown to comprise, in addition to diploids, allotriploids and allotetraploids (Zarrei et al. http://2012.botanyconference.org/engine/search/index.php?func=detail&aid=536 and unpubl. data).

In order to increase our sample for molecular studies we have supplemented field collections of leaf tissue and herbarium vouchers with tissue removed from existing specimens in the ROM Green Plant Herbarium. Historical records of the distribution of *Crataegus monogyna* were collected from five herbaria across Canada (TRT, MTMG, MT, QFA and UBC). Online databases of Canadian and U.S. herbaria used included ACAD, the Invader Database System of the University of Montana (which contains information for five northwestern states: Idaho, Montana, Oregon, Washington, Wyoming), OSC, and WTU. Distribution maps were prepared from specimen locality data using SimpleMappr (Shorthouse 2010). Names of *Crataegus* sections and series used here follow those published by VASCAN (Brouillet et al. 2010), and are accepted names sensu FNA Ed. Comm. in prep.

**Morphology.** For this study we concentrated on capturing and analyzing leaf shape data, as described elsewhere (Dickinson et al. 2008). Many previous studies of hybridization involving *Crataegus monogyna* (Bradshaw 1953; Byatt 1975; Love and Feigen 1978), and of leaf shape variation in *Crataegus* generally (e.g. El-Gazzar 1980; Phipps and O’Kennon 2007), have attempted to quantify leaf lobing by means of a ratio of two measurements, *x* and *y*, where *x* is the distance from the tip of a lobe (usually the most basal one) to the deepest point of the sinus between that lobe and the adjacent one above it, and *y* is a measure of leaf size, usually the parallel distance from the tip of the lobe to the midrib. This approach can be effective when comparisons involve only leaves that have some degree of lobing (e.g. studies of hybridization between *Crataegus monogyna* and *Crataegus laevigata* in Europe, or of the lobed leaves of many species belonging to North American *Crataegus* sect. *Coccineae*, such as *Crataegus punctata*). However, when lobing is absent altogether the necessary landmarks (lobe tip, deepest point of the sinus) are absent, and the distance *x* is undefined or is set to zero (Love and Feigen 1978). In this case, a better approach is to carry out multivariate analyses of additional measurements of leaves and other organs (Wells and Phipps 1989), or to quantify variation in the leaf outline as a whole. Elliptic Fourier coefficients obtained from digitized leaf outlines captured using MorphoSys (Meacham and Duncan 1991), or the Fourier amplitudes derived from them, provide a useful method for doing just this (Dickinson et al. 2008; McLellan and Endler 1998; Rohlf and Archie 1984).

Leaf outline data were collected from two overlapping samples: (1) short shoot leaf spectra (Dickinson and Phipps 1984) collected from a random sample of individuals at the Cogswell-Foster Preserve (comprising one *Crataegus suksdorfii*, seven *Crataegus monogyna*, and 12 putative hybrids), and (2) leaves on herbarium specimens from the Cogswell-Foster Preserve and other locations in the Pacific Northwest. In the latter the attempt was made to sample the leaf shape variation seen in *Crataegus suksdorfii* as widely as possible. In both cases, variation in the shape of the leaf blade (i.e. excluding the petiole) was summarized by means of 39 Fourier amplitudes, and displayed by means of principal components analysis.

For each leaf outline we also obtained the area (*A*) and perimeter (*P*), so as to calculate the inverse of the dissection index described by Kincaid and Schneider (1978), i.e. *inv(D.I.)* = 2(*A*π)^1/2^/*P*, a parameter that has an upper bound of one for a perfect circle regardless of size, and approaches zero as the length of the perimeter increases with increased lobing of the outline (Dickinson 2003; Dorken and Barrett 2004). In addition to outline data we made linear measurements with which to index overall leaf shape: *X*, leaf blade length above the widest point; *Y*, leaf width; and *Z*, leaf blade length below the widest point (Marshall 1978). On some of the flowering specimens in our sample we collected additional data on stamen number, style length and style number (in fruiting specimens, equivalently, pyrene number), and stigma width, in order to compare these with data collected by others from the introduced species and *Crataegus punctata*. After transformation to a common [0,1] range these data were also summarized using principal components analysis. Analyses of variance were carried out on selected measurements. All data analyses described above were carried out using the R environment for statistical computing (R Core Team 2013). Significance of individual principal component axes was evaluated using the broken-stick criterion (Frontier 1976) with the help of R function *evplot* (Borcard et al. 2011).

**Molecular methods.** Four DNA barcodes (*rbcL*, *matK*, *trnH*-*psbA*, and ITS2; CBOL Plant Working Group 2009; Chase et al. 2007; Hollingsworth et al. 2011) were generated directly from genomic DNA for a worldwide sample of *Crataegus* (Dickinson et al. http://2011.botanyconference.org/engine/search/720.html; Zarrei et al. unpubl. data). The plastid origin of the markers was used to establish the maternal parentage of the hybrids. DNA was extracted and amplified from leaf tissue of individuals representing the two hybrids and their parent species (Table 2) using Canadian Centre for DNA Barcoding (CCDB) protocols (Ivanova et al. 2011; Kuzmina and Ivanova 2011a, b). This sample overlapped partially with the cloned ITS2 one (below), and provided an additional two *Crataegus suksdorfii*, 10 *Crataegus monogyna*, and five *Crataegus punctata* individuals, as well as one more of each of the two hybrids (Table 2).

We also analyzed data from another project (Zarrei et al. http://2012.botanyconference.org/engine/search/index.php?func=detail&aid=536 and unpubl. data) in which ITS2 was cloned for a sample of individuals that included 14 *Crataegus suksdorfii*, four *Crataegus monogyna*, three *Crataegus punctata* and two each of the two hybrids (Table 3). Methods for extracting total genomic DNA, marker amplification, cloning, DNA sequencing, and collapsing original sequences to unique sequences (ribotypes) are described elsewhere (Zarrei et al. http://2012.botanyconference.org/engine/search/index.php?func=detail&aid=536 and unpubl. data). Here we report on analyses of a total of 160 ribotypes (Table 3). A recombination test was performed using RDP4 Beta 4.14 (Martin et al. 2010). The Neighbor-Net analysis (Bryant and Moulton 2004) was undertaken using SplitsTree v.4.12.3 (Huson and Bryant 2006) to visualize incompatible splits in the network from uncorrected p-distances calculated with MEGA5 (Tamura et al. 2011). Bootstrap support (BS) was estimated using 1,000 bootstrap pseudoreplicates (Felsenstein 1985) implemented in SplitsTree.

**Flow cytometry.** Flow-cytometric methods for quantifying nuclear DNA in embryo and endosperm followed Talent and Dickinson (2007a). Embryo DNA amounts of 1.48–1.70 pg were taken to indicate diploids, and an endosperm to embryo ratio of approximately 1.5 was taken to indicate sexual reproduction with meiosis.

## Results and discussion

**Figure 1.**
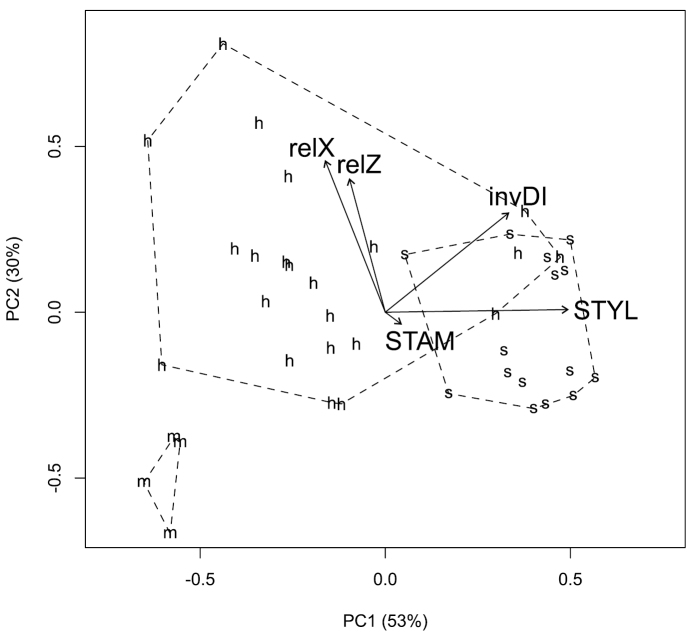
Principal components analysis biplot for five morphometric descriptors averaged for each of 41 *Crataegus* herbarium specimens from the Cogswell-Foster Preserve and other locations in the Pacific Northwest (*Crataegus suksdorfii* (s), *Crataegus monogyna* (m), and the putative hybrid, *Crataegus ×cogswellii* (h)): *relX*, leaf length above the widest point, scaled by the width; *relZ*, leaf length below the widest point, scaled by the width; *invDI*, inverse dissection index = 2(*A*π)^1/2^/*P*, where *A* is the leaf area and *P* is the leaf perimeter; *STAM*, number of stamens per flower; *STYL*, number of styles per flower. Both axes shown account for significant portions of the total variance according to the broken-stick criterion (Frontier 1976).

**Figure 2.**
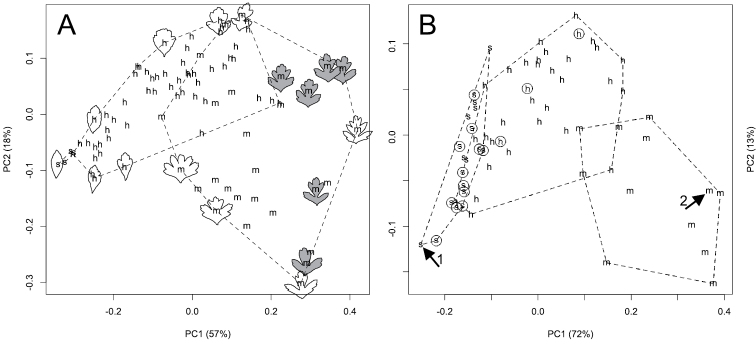
**A** Principal components analysis of 39 Fourier amplitudes for 86 subterminal short shoot leaves from 20 *Crataegus* individuals at the Cogswell-Foster Preserve in Linn Co., Oregon (one *Crataegus suksdorfii* (s), seven *Crataegus monogyna* (m), and 12 putative hybrids (h), *Crataegus ×cogswellii*). Leaf outlines illustrate the shape contrasts responsible for the ordination: in grey, six subterminal leaves from short shoots of a single individual (OR1–8) **B** Principal components analysis of 39 Fourier amplitudes averaged for leaves sampled regardless of position on short shoots of 64 herbarium specimens from the Cogswell-Foster Preserve and (circled points) other locations in the Pacific Northwest (Table 1). In both A and B the two PCA axes shown are significant according to the broken-stick criterion (Frontier 1976). In **B** arrowed point 1 represents the single individual of *Crataegus suksdorfii* for which individual leaves are represented in **A**, while arrowed point 2 represents the averaged data for the six leaves of *Crataegus monogyna* shown in grey in **A.**

**Morphology.** Despite differences in sample size, the Pacific Northwest hybrid, *Crataegus ×cogswellii*, appears more variable than either of its putative parents, *Crataegus monogyna* or *Crataegus suksdorfii* (Fig. 1). The hybrid is clearly intermediate with respect to both leaf lobing (the inverse Dissection Index; Fig. 1) and style number (STYLE; Fig. 1). Principal components analyses of leaf outlines from Pacific Northwest *Crataegus monogyna*, *Crataegus suksdorfii*, and their putative hybrid, demonstrate variation in leaf shape both within and between these three entities (Fig. 2A, B). The first principal component reflects the contrast between the unlobed leaves of *Crataegus suksdorfii* and the markedly lobed ones of *Crataegus monogyna*, as well as the intermediacy of the hybrid (Fig. 2A, B), much as illustrated earlier by Love and Feigen (1978; their Fig. 3), and by Wells and Phipps (1989) for the Ontario hybrid and its parents (their Fig. 4). The second principal component reflects variation in the relative overall lengths and widths of the leaf outlines (Fig. 2A).

**Figure 3.**
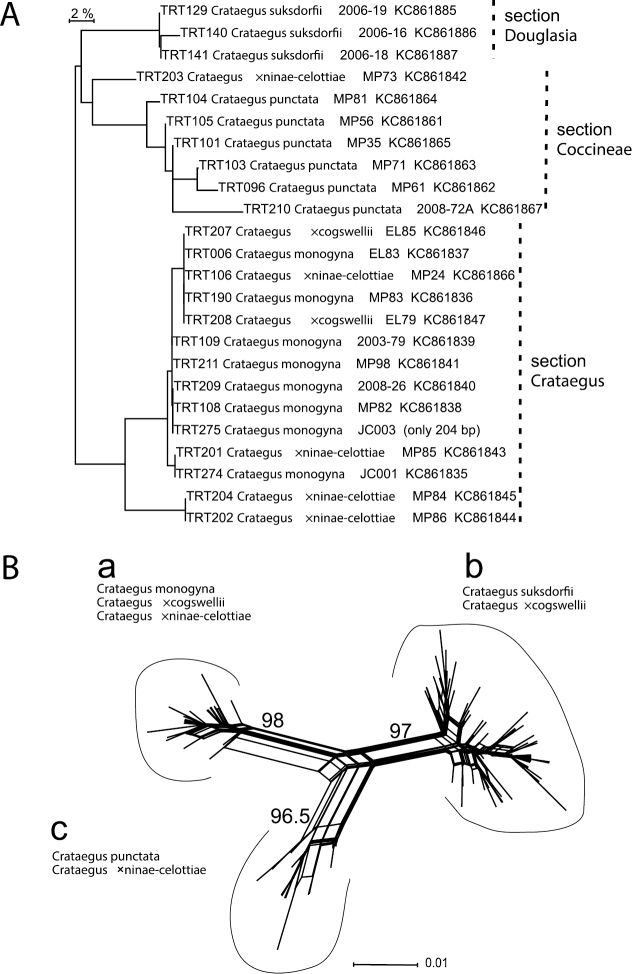
**A** Neighbor-joining tree calculated by BOLD for ITS2 DNA barcode sequences amplified directly from genomic DNA (labels include corresponding collector and GenBank number; see dx.doi.org/10.5883/DS-CRATMONO and Table 1 for details). Dashed lines indicate the sectional affinity of the sequences **B** The corresponding Neighbor-Net network for the cloned ITS2 sequences has three branches representing: (**a**) ribotypes from individuals of *Crataegus monogyna*, and from its hybrids with both *Crataegus suksdorfii* and *Crataegus punctata*; (**b**) ribotypes from individuals of *Crataegus suksdorfii* and *Crataegus ×cogswellii*; and (**c**) ribotypes from individuals of *Crataegus punctata* and *Crataegus ×ninae-celottiae* (Table 3). The numbers shown are the % bootstrap support for each of the three branches.

**Figure 4.**
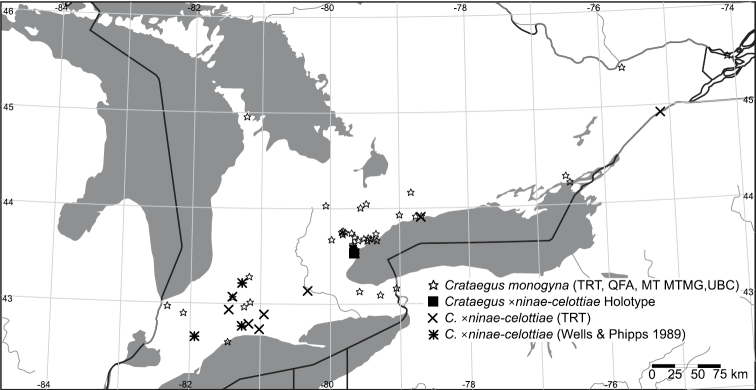
Geographic distribution of *Crataegus ×ninae-celottiae* K.I. Chr. & T.A. Dickinson nothosp. nov. and *Crataegus monogyna* in Ontario. Filled square, holotype of *Crataegus ×ninae-celottiae*; Crosses, TRT specimens of *Crataegus ×ninae-celottiae*; asterisks, *Crataegus ×ninae-celottiae* specimens cited by Wells and Phipps (1989); stars, specimens of *Crataegus monogyna* in MT, MTMG, QFA, TRT, and UBC. *Crataegus punctata* occurs throughout the region depicted (Phipps and Muniyamma 1980; this paper also maps additional records for *Crataegus monogyna*).

**DNA barcode loci.** Analyses of both the directly sequenced and the cloned ITS2 ribotypes demonstrate the parentage of both putative hybrids (Fig. 3; Table 3); no signs of recombination were detected in the cloned ITS2 dataset. ITS2 sequences from the hybrids resemble either *Crataegus monogyna* or one of the native North American species. The way in which both parental ribotypes are maintained in each of the hybrids examined here is probably due to how recently the hybrids have been formed: less than 200 years ago in the case of the Ontario hybrids (Douglas 1914; Kirk 1819; Provancher 1863), and less than 100 years ago in the case of the Oregon ones (the earliest specimen of *Crataegus monogyna* was collected in 1914 in Douglas Co. Oregon; Phipps 1998). These time periods are evidently too short for genome homogenization (concerted evolution) to have taken place, even in diploids reproducing sexually. Our small sample of seed from the hybrids (Table 4) parallels earlier results (Talent and Dickinson 2007a) showing diploidy and sexual reproduction in both parental taxa.

**Table 4.** Flow-cytometric results from seeds of the two described *Crataegus* nothospecies. The ratios shown for endosperm and embryo nuclear DNA contents are well within the ranges observed for sexually reproducing *Crataegus monogyna* (Talent unpubl. data) and diploid *Crataegus suksdorfii* (Lo et al. 2013).

Taxon / TRT accession / site / collection	Total number seeds	Mean embryo DNA	Mean endosperm:embryo ratio (number of seeds)
*Crataegus ×ninae-celottiae*			
ON45 2002-13 (TRT00000406)	2	1.58 pg	1.56 (2)
ON31 EH52 (TRT00002256)	1	1.67 pg	1.53 (1)
*Crataegus ×cogswellii*			
OR1 EL-79 (TRT00002657)	3	2.08 pg	1.58 (1)
OR1 2009-36 (TRT00002568)	1	1.87 pg	1.60 (1)

Only two of the three chloroplast genome barcode loci showed sufficient variation for individuals from *Crataegus* section *Crataegus* to be distinguished from ones belonging to either *Crataegus* section *Coccineae* or *Crataegus* section *Douglasia* (Table 2). Sequence data from both *rbcL-a* and the *trnH-psbA* spacer region showed the same two clusters, *Crataegus* sections *Coccineae* and *Douglasia* (Cluster 1), and *Crataegus* sect. *Crataegus* (Cluster 2; Table 2). The way in which the hybrids fell into one of these clusters or the other demonstrates that, with one exception, *Crataegus monogyna* is the female parent of the Ontario hybrids with *Crataegus punctata* studied here, while *Crataegus suksdorfii* is the female parent of the Pacific Northwest hybrids.

These results corroborate earlier observations based on seed-set in artificial crosses between the parent species (Love and Feigen 1978; Wells and Phipps 1989). In reciprocal pollinations seed set was greatest (32–34%) when *Crataegus monogyna* stigmas received pollen from *Crataegus punctata* (Wells and Phipps 1989). Fruit set was most successful when *Crataegus monogyna* pollen was applied to the stigmas of *Crataegus suksdorfii* flowers (mean 42%, range 25–73%, compared to a 29% mean fruit set by *Crataegus suksdorfii* with open pollination; Love and Feigen 1978). However, all reciprocal crosses between *Crataegus monogyna*, *Crataegus suksdorfii*, and their hybrid yielded seeds (R. M. Love, personal communication).

Our use of data from DNA barcoding is not a test of the value of DNA barcoding in *Crataegus*, as this is discussed elsewhere (Dickinson et al. http://2011.botanyconference.org/engine/search/720.html; Zarrei et al. unpubl. data). Rather, we have taken advantage of our barcode sequence data from individuals unequivocally identifiable as *Crataegus monogyna*, *Crataegus punctata*, *Crataegus suksdorfii* and their hybrids in order to use sequence similarity to inform us about the hybridization process.

**Figure 5.**
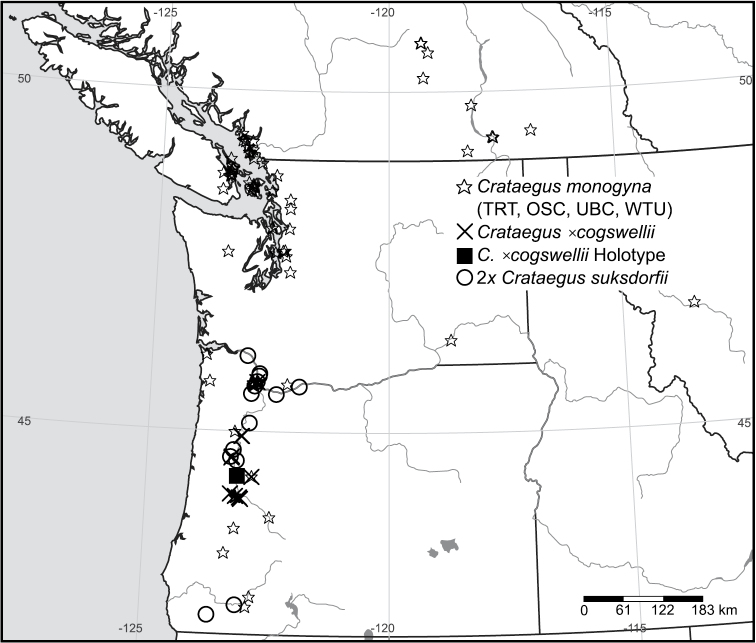
Geographic distribution of *Crataegus ×cogswellii* K.I. Chr. & T.A. Dickinson nothosp. nov. and its parental species in the Pacific Northwest. Filled square, holotype of *Crataegus ×cogswellii*; crosses, TRT specimens of *Crataegus ×cogswellii*; circles, diploid *Crataegus suksdorfii*; stars, *Crataegus monogyna* (specimens in OSC, TRT, UBC, and WTU).

**Hybridization.** Since its introduction to North America during the late 18^th^ and the 19^th^ centuries (Kirk 1819; Provancher 1863; Douglas 1914), first on the east coast and then on the west, *Crataegus monogyna* has become widely naturalized in the U.S.A. (EDDMapS 2013) and Canada (Phipps and Muniyamma 1980; Phipps 1998; Lin 2009). Nevertheless, except for isolated occurrences in northern Delaware and adjacent Pennsylvania, as well as in Kentucky, Utah, and the San Francisco Bay area in California, *Crataegus monogyna* in North America is not found south of 40°N latitude. In Ontario, *Crataegus punctata* appears to be the only native diploid with a similarly late flowering period that is also frequently sympatric with *Crataegus monogyna* (Fig. 1 in Campbell et al. 1991; Fig. 4). *Crataegus suksdorfii* is the only native hawthorn in the Pacific Northwest known to include diploid individuals, and these are restricted to Oregon and adjacent California and Washington, west of the Cascades (Fig. 5; Lo et al. 2013). Where they co-occur, diploid *Crataegus suksdorfii* and *Crataegus monogyna* flower at the same time, the latter species much more abundantly than the former (Love and Feigen 1978).

*Crataegus monogyna* may never have been commonly planted in boundary hedges in Canada as it was in Europe. Fences and hedges appear to have been only rarely constructed in 17th Century Canada by European settlers to confine ruminant animals (Greer 2012); the animals were instead fed indoors, but allowed to roam the arable land for a short season after harvest, confined by the wall of surrounding forest. To this day, the hawthorn commonly growing along Ontario fence lines consists of native species, perhaps naturally occurring there. In Ontario forests we often encounter remnants of zig-zag post-and-rail fences, and these had the advantage over a hedge that they could be rapidly constructed as needed to mark property boundaries or to keep animals out of particular areas. In the United States hedging had its advocates in the early nineteenth century, but one of these described the superiority of native species like *Crataegus crus-galli* (“cockspur” or “Newcastle thorn”) and *Crataegus marshallii* (“parsley-leaved” or “Virginia thorn”) over introduced *Crataegus monogyna* (Kirk 1819; “to sow or plant without fencing, would (in this country) be a useless labour”).

Flow cytometry of seeds from both hybrids was consistent with diploid embryos and triploid endosperm, except that the embryos from *Crataegus ×cogswellii* show slightly higher than diploid measurements, higher than the 1.39–1.66 pg measurements previously obtained from leaf data (Table 4; Talent and Dickinson 2005). Whether the seeds involved would have germinated is unknown, but in contrast to the large healthy looking seeds from *Crataegus ×ninae-celottiae*, those from *Crataegus ×cogswellii* had smaller embryos and were variously misshapen. We noted that some individual trees of *Crataegus ×cogswellii* have a high degree of parthenocarpy—completely seedless fruit—and the seeds we collected may therefore have been supernumerary to any strongly viable seeds. We can only state that *Crataegus ×cogswellii* apparently carries out both meiosis and fertilization, as expected of other diploid *Crataegus* (Table 4; Talent and Dickinson 2007b).

In her examination of hybridization between *Crataegus punctata* and *Crataegus monogyna* in Ontario, Purich (2005) found that the styles of *Crataegus punctata* are significantly longer than those of *Crataegus monogyna* (mean_mono_ = 4.1 mm; mean_punc_ = 7.3 mm; sample sizes 5/52 and 7/116, individuals/styles). Differences between the two species in pollen grain diameter, hence volume, are not significant (Purich 2005). No such difference in style length is present when comparing *Crataegus monogyna* and *Crataegus suksdorfii*. These results suggest that in Ontario, at least, the longer styles of *Crataegus punctata* could act as a barrier to the successful penetration of *Crataegus punctata* ovules by pollen tubes from *Crataegus monogyna* pollen grains (Table 2). With style lengths and pollen grain diameters in *Crataegus monogyna* and *Crataegus suksdorfii* similar (Dickinson unpublished data), it may be that the more abundant flower production of *Crataegus monogyna* (Love and Feigen 1978) contributes to its role as the predominant pollen parent of *Crataegus ×cogswellii*. The exception to the summary above (TRT203 in Table 2; *Crataegus punctata* as the maternal parent) reflects the way in which differences in style length likely act to influence the direction of hybridization in a probabilistic rather than an absolute way.

## Taxonomy

### *Crataegus* nothosect. *Coccitaegus* K.I. Chr. & T.A. Dickinson nothosect. nov. (*Crataegus* sect. *Coccineae* × sect. *Crataegus*)

*Crataegus* nothoser. *Punctaegus* K.I. Chr. & T.A. Dickinson **nothoser. nov.** (*Crataegus* ser. *Crataegus* × ser. *Punctatae*)

**Figure 6.**
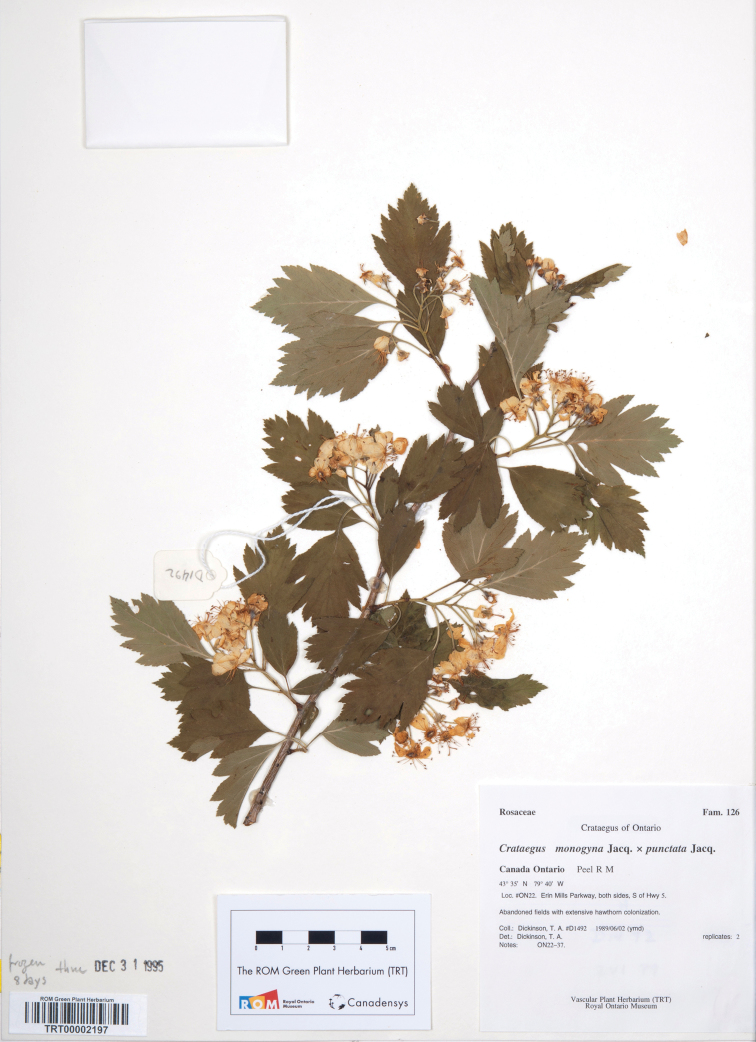
Holotype of *Crataegus ×ninae-celottiae* K.I. Chr. & T.A. Dickinson nothosp. nov. (♀*Crataegus monogyna* × ♂*Crataegus punctata*): TRT00002197, CANADA, Ontario, Peel R M, loc. ON22, Don Gould Park and E side of Erin Mills Parkway, 43°35'N, 79°40'W, abandoned fields with extensive hawthorn colonization, 2 Jun 1989, Dickinson D1492.

*Crataegus ×ninae-celottiae* K.I. Chr. & T.A. Dickinson **nothosp. nov.** (Fig. 6). – Type: CANADA, Ontario, Peel R M, Don Gould Park and E side of Erin Mills Parkway (ON22), 43°31.960'N, 79°39.591'W, woodlot and fields with extensive hawthorn colonization, 2 Jun 1989, Dickinson D1492 (holotype TRT00002197!; isotype S!) (♀*Crataegus monogyna* × ♂*Crataegus punctata*)

*Ramunculi pubescenti vel glabri. Folia distalia ramorum fertilium non profunde quinque-undecim-partita, 30–55 mm longa, 16–38 mm lata, nervi supra profunde impressi; stipulae caducae, 3–4 mm longae, plusminusve denticulatae. Inflorescentiae 5–17-florae, laxae, pubescentae; bracteae caducae, plusminusve denticulatae. Sepala integra, rarius sparsim glandulosa, post anthesin reflexa. Fructus 9–12 mm longus, 12–14 mm latus, ruber vel aurantiacus; pulpa lutea, mitis et succida; pyrenae 2–3, ventraliter sulcatae vel foveatae*.

**Remarks.** Shrub or tree up to ca. 6 m tall. Twigs of the current year densely to sparsely hairy or glabrous, hairs appressed to patent, straight or slightly curly; twigs of the previous year pale grey or ash-grey; aphyllous thorns 0.5–2 cm long, stout, straight; spine-tipped, leaf- and dwarf-shoot-bearing branchlets lacking. Leaf blades ovate, obovate or elliptical, acute at apex, attenuate, cuneate or rounded at base, shallowly or deeply and regularly lobed, lobes with an acute apex, basal pair of veins convergent, straight or slightly divergent, intercalary veins running to the sinuses partly present, upper surface with ± deeply impressed veins at maturity, dull or lustrous bright or dark green, sparsely hairy and often becoming glabrous except along the veins, hairs appressed or semi-patent; lower surface dull, pale green, sparsely hairy throughout or only along the major veins and in the vein axils, hairs appressed or semi-patent; margin regularly crenate-serrate or serrate, teeth minutely glandular, glands less than 0.1 mm; petiole eglandular, narrowly winged in upper part. Subterminal leaf blade of flowering shoots 30–55 mm long, 16–38 mm wide, shallowly and regularly lobed, lobes 2–5 pairs, basal pair extending 0.2–0.4 times the width of lamina to midrib, each lobe with 6–11 teeth, basal pair of sinuses in apical 1/4 to basal 1/3 of lamina; petiole 6–20 mm long; stipules caducous, membranous or herbaceous, 4–8 mm long, irregularly or regularly glandular-denticulate, with 20–30 teeth. Leaf blades of elongate shoots 35–45 mm long, 25–35 mm wide, shallowly or deeply and regularly lobed, lobes 3–5 pairs, basal pair extending 0.2–0.6 times the width of lamina to midrib, each lobe with 4–11 teeth, basal pair of sinuses in basal 1/2–1/3 of lamina; petiole 8–12 mm; stipules caducous, herbaceous, ca. 6 mm long, regularly glandular denticulate-serrate, with ca. 15 teeth. Inflorescence 3–4 cm long, lax, corymbose, 5–17-flowered, densely to sparsely hairy, hairs appressed, semi-patent or patent, straight or slightly curly; pedicels 3–18 mm, densely to sparsely hairy, hairs appressed, semi-patent or patent, straight or slightly curly; bracts caducous, membranous or herbaceous, 3–4 mm long, 0.2–0.4 mm wide, linear-lanceolate, 10–15 times as long as wide, irregularly glandular-denticulate, with 5–7 teeth. Hypanthium 3–4 mm long, densely to sparsely hairy, hairs appressed, semi-patent or patent, straight or slightly curly; sepals 2–4 mm long, 1.5–2 mm wide, triangular-lanceolate or triangular, 1–2.7 times as long as wide, entire or rarely irregularly and minutely glandular-serrate, teeth 0–2, apex acute or obtuse; petals 6–7 mm long and wide; stamens 18–20, anthers 1–1.2 mm long, pink or purple; styles 2–3; hypostyle pilose. Fruit 9–12 mm long, 8–12 mm in diameter, 1.0–1.1 times as long as wide, globose, broadly ellipsoidal or obovoid, ± lustrous, red or orange, punctate with small, pale brown lenticels, up to ca. 0.2 mm in diameter, sparsely hairy, crowned by the persistent, reflexed sepals; calyx tube indistinct, ca. 0.5 mm long, 3–4 mm wide; flesh yellowish, hard and mealy; pyrenes 2–3, ventro-laterally smooth; hypostyle pilose.

**Phenology.** Flowering in May–June. Fruiting in August–September.

**Reproductive biology.** Sexual. 2*n* = 2*x* (2*n* = 34? Muniyamma and Phipps 1979; Talent and Dickinson 2005); diploid embryos and triploid endosperm.

**Distribution.** Eastern Canada. Ontario (Fig. 4).

**Etymology.** *Crataegus ×ninae-celottiae* honors Nina Celotti (1971–1995), who studied the pollination pathway of the two parent species, *Crataegus punctata* and *Crataegus monogyna*.

**Similar taxa.** *Crataegus ×ninae-celottiae* differs from *Crataegus monogyna* in: spine-tipped, leaf- and dwarf-shoot-bearing branchlets lacking; leaf blades with ± deeply impressed veins above; subterminal leaf blade of flowering shoots shallowly lobed, lobes 2–5 pairs (not ± deeply lobed and lobes 1–3 pairs); stipules caducous, often membranous, irregularly or regularly glandular-denticulate, with 20–30 teeth (not ± persistent, herbaceous and ± entire); styles and pyrenes 2–3 (not 1–(2)); fruit often orange, punctate with pale brown lenticels up to ca. 0.2 mm in diameter.

*Crataegus ×ninae-celottiae* differs from *Crataegus punctata* in: aphyllous thorns shorter, 0.5–2 cm long (not 2–5 cm long); leaf blades regularly lobed almost to the base (not unlobed or shallowly lobed towards apex), intercalary veins running to the sinuses sometimes present; subterminal leaf blade of flowering shoots usually smaller, up to ca. 55 mm long, and veins 2–5 pairs (not up to ca. 85 mm and veins 6–10 pairs); stipules often herbaceous and irregularly glandular-denticulate; sepals shorter, 2–4 mm long, and wider, 1–2.7 times as long as wide (not 3–7 mm long and 2–4.7 times as long as wide); styles and pyrenes 2–3 (not 3–5); fruit usually smaller, up to ca. 12 mm long and in diameter (not up to ca. 15 mm long and in diameter) and less distinctly punctate with smaller lenticels up to ca. 0.2 mm in diameter (not up to ca. 0.4 mm in diameter).

*Crataegus ×ninae-celottiae* was studied by Phipps and Muniyamma (1980) and by Wells (Wells and Phipps 1989), who documented the intermediacy of the hybrid relative to its parents in leaf, thorn, flower, and fruit characteristics. In addition, paper chromatography was used to compare phenolic profiles of the three entities, which also demonstrated intermediacy. These results have been corroborated using thin layer chromatography (Harris 2001). Both parents and the hybrid are diploids (*x* = 17, as in other Maleae; Muniyamma and Phipps 1979; Talent and Dickinson 2005), and both parents are highly pollen fertile (stainability > 80%). Pollen stainability in the hybrid was found to be variable (27–97%, mostly in the range 60–80%; Purich 2005).

**Specimens examined, paratypes** (in bold, specimens in Tables 2–4). **CANADA, Ontario:** Peel Co., City of Mississauga, Don Gould Park and E side of Erin Mills Parkway (ON22), 1989-06-02, Dickinson D1480 (TRT00000408!); 1989-06-02, Dickinson D1482 (TRT00000407!); 1989-05-31, Dickinson D1485 (TRT00000409!); 2000-05-19, Talent NT-03 (TRT00002306!); 2011-05-28, Christensen & Dickinson s.n. (TRT00024869!). Middlesex Co., Denfield Twp., SE corner Denfield Side Road and Ilderton Road (ON31), 2001-05-17, **Harris & Dickinson EH-52 (TRT00002256!)**; 2001-05-17, Harris & Dickinson EH-54 (TRT00002257!); 2002-07-30, Talent & Dickinson EH52 (TRT00000405!). Durham R.M., Bowmanville, floodplain of Bowmanville Creek (ON45), 2002-09-30, **Dickinson & Nguyen 2002-13 (TRT00000406!)**, 2004-06-03, **Purich 85 (TRT00002250!)**, 2004-06-03, **Purich 86 (TRT00002251!)**.

### *Crataegus* nothosect. *Crataeglasia* K.I. Chr. & T.A. Dickinson nothosect. nov. (*Crataegus* sect. *Crataegus* × sect. *Douglasia*)

*Crataegus* nothoser. *Crataeglasianae* K.I. Chr. & T.A. Dickinson **nothoser. nov.** (*Crataegus* ser. *Crataegus* × ser. *Douglasianae*)

**Figure 7.**
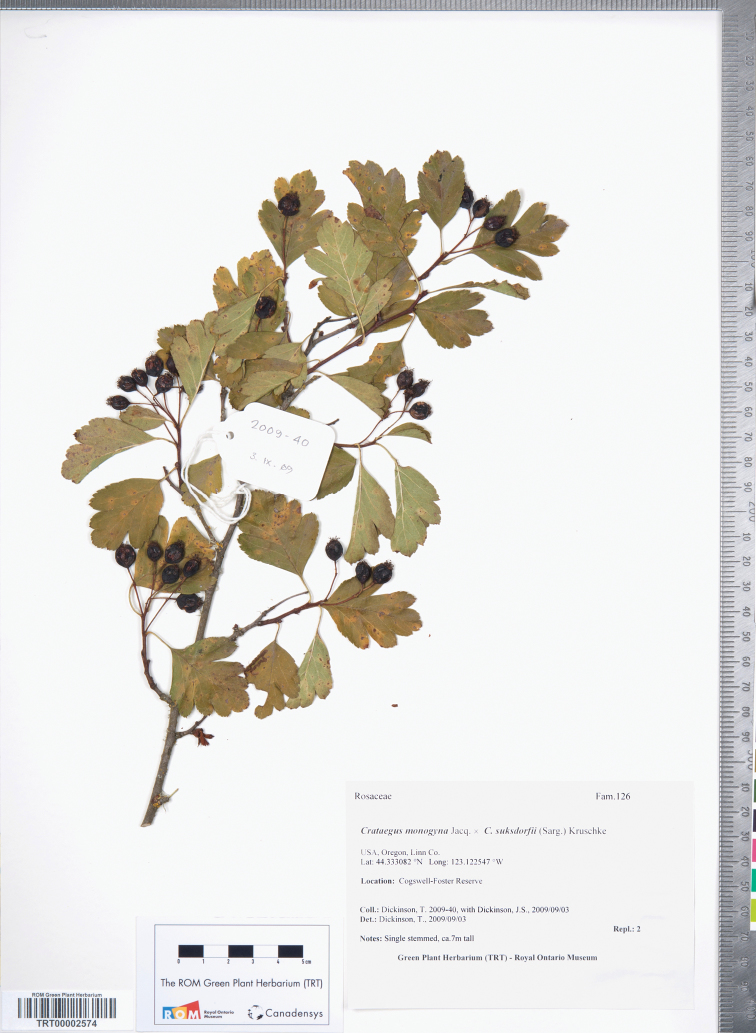
Holotype of *Crataegus ×cogswellii* K.I. Chr. & T.A. Dickinson nothosp. nov. (♀*Crataegus suksdorfii* × ♂*Crataegus monogyna*): TRT00002574, U.S.A., Oregon, Linn Co., Cogswell-Foster Preserve, 44.333082°N, 123.122547°W, 3 Sep 2009, Dickinson & Dickinson 2009-40.

*Crataegus ×cogswellii* K.I. Chr. & T.A. Dickinson **nothosp. nov.** (Fig. 7.). – Type: U.S.A., Oregon, Linn Co., Cogswell-Foster Preserve, 44°19.985'N, 123°7.353'W, 3 Sep 2009, Dickinson & Dickinson 2009-40 (holotype TRT00002574!; isotype TRT). (♀*Crataegus suksdorfii* × ♂*Crataegus monogyna*)

*Ramunculi glabri vel rarius sparsim villoso-lanati. Folia distalia ramorum fertilium quinque-novem-partita, rarius integra, 25–70 mm longa, 15–50 mm lata; stipulae caducae, 4–8 mm longae, plusminusve denticulatae. Inflorescentiae 4–25-florae, laxae, glabrae vel rarius villoso-lanatae; bracteae caducae, plusminusve denticulatae. Sepala integra vel rarius sparsim glandulosa, post anthesin reflexa. Fructus 9–12 mm longus, 12–14 mm latus, lampro-atro-purpureus vel anthracinus; pulpa lutea, mitis et succida; pyrenae 2–5, ventraliter sulcatae vel foveatae*.

**Remarks.** Shrub or tree up to ca. 12 m tall. Twigs of the current year glabrous, rarely sparsely villous-lanate; twigs of the previous year dark reddish-brown or pale- or dark-grey; aphyllous thorns 0.5–2 cm long, stout, straight or slightly recurved; spine-tipped, leaf- and dwarf-shoot-bearing branchlets lacking, rarely present. Leaf blades broadly or narrowly obovate, ovate, rhombic-ovate or elliptical, acute at apex, attenuate, cuneate or rounded at base, deeply or shallowly and regularly lobed, rarely some leaves unlobed, lobes with an acute or obtuse apex, basal pair of veins divergent or straight, intercalary veins running to the sinuses usually present; upper surface dull, dark green, sparsely hairy especially along the veins, hairs appressed or semi-patent; lower surface dull, pale green, villous in the vein axils and occasionally along the major veins; margin regularly and ± coarsely or finely crenate-serrate or serrate, teeth eglandular or minutely glandular, glands less than 0.1 mm; petiole eglandular or rarely sparsely glandular, narrowly winged in upper part. Subterminal leaf blade of flowering shoots 25–70 mm long, 15–50 mm wide, deeply or shallowly and regularly lobed, rarely unlobed, lobes (0–)2–4 pairs, basal pair extending 0.2–0.8 times the width of lamina to midrib, each lobe with 5–18 teeth, basal pair of sinuses in apical 1/3 to basal 1/3 of lamina; petiole 5–15 mm long; stipules persistent or caducous, herbaceous, 5–12 mm long, irregularly or regularly glandular denticulate-serrate or serrate, with 4–30 teeth. Leaf blades of elongate shoots 40–90 mm long, 30–50 mm wide, deeply or shallowly and regularly lobed, lobes 1–4 pairs, basal pair extending 0.4–0.9 times the width of lamina to midrib, each lobe with 7–20 teeth, basal pair of sinuses in basal 1/2–1/5 of lamina; petiole 10–20 mm; stipules persistent or caducous, herbaceous, 6–14 mm long, regularly glandular denticulate-serrate or serrate, with 15–30 teeth. Inflorescence 2.5–5 cm long, lax, corymbose, 4–25-flowered, glabrous, rarely sparsely villous-lanate; pedicels 4–11 mm, glabrous, rarely sparsely villous-lanate; bracts caducous or very rarely persistent, membranous or herbaceous, 3–10 mm long, 0.2–2.5 mm wide, linear-lanceolate, 4–10 times as long as wide, regularly glandular-serrate or ± irregularly glandular-denticulate, with 4–22 teeth. Hypanthium 2–3 mm long, glabrous or rarely sparsely villous-lanate; sepals 1–2.5 mm long, 1.5–2 mm wide, triangular, 0.5–1.7 times as long as wide, entire or very rarely irregularly and minutely glandular-serrate, teeth 0–2, apex acute or obtuse; petals 4–6 mm long and wide; stamens 18–20, occasionally vestigial, anthers 0.6–1 mm long, purple; styles 2–5; hypostyle pilose. Fruit 6–9 mm long, 6–8 mm in diameter, 1–1.2 times as long as wide, globose-subglobose or broadly ellipsoidal, epruinose, ± lustrous, blackish purple or black, glabrous-subglabrous, crowned by the persistent, reflexed sepals; calyx tube indistinct, 0.4–1 mm long, 3.5–4.5 mm wide; flesh yellowish, soft and juicy; pyrenes 2–5, irregularly ventro-laterally pitted; hypostyle pilose.

**Phenology.** Flowering in April–May. Fruiting in September. Some individuals strongly parthenocarpic.

**Reproductive biology.** Sexual. 2*n* = 2*x* [≈ 34] (Talent and Dickinson 2005); diploid embryos and triploid endosperm. Chromosome number: 2*n* = 2*x* = 34, estimated from flow cytometry data (Table 4); chromosome counts have not been made.

**Distribution.** Northwestern U.S.A.; western Oregon (Figure 5); potentially present in adjacent northwestern California and southwestern Washington where the parent species are sympatric.

**Etymology.** *Crataegus ×cogswellii* honours the Cogswell family, and Mr. and Mrs. Lee Foster, of Halsey, Oregon. In 1872 John Cogswell, Mrs. Foster’s grandfather, purchased the land that the Fosters gave to the Oregon Nature Conservancy as the Cogswell-Foster Preserve (Lopez 1971), and at which *Crataegus ×cogswellii* has been most intensively studied (Love and Feigen 1978).

**Similar taxa.** *Crataegus ×cogswellii* differs from *Crataegus monogyna* in: leaf- and dwarf-shoot-bearing branchlets usually lacking; stipules of leaves of flowering shoots irregularly or regularly glandular denticulate-serrate or serrate (not ± entire); styles and pyrenes 2–5 (not 1–(2)); fruit blackish purple or black (not bright or dark red).

*Crataegus ×cogswellii* differs from *Crataegus suksdorfii* in: twigs of the current year occasionally sparsely villous-lanate; leaf- and dwarf-shoot-bearing branchlets occasionally present; leaf blades usually deeply or shallowly and regularly lobed, intercalary veins running to the sinuses usually present; inflorescence, pedicels and hypanthia occasionally sparsely villous-lanate; hypostyle pilose (not glabrous or sparsely pilose).

**Specimens examined, paratypes** (in bold, specimens in Tables 2–4). **U.S.A., Oregon:** Columbia Co., Sauvie Island (OR11), 2003-06-14, Zika 18482 (TRT00002651!); 2005-08-31, Lo & Dickinson 103.2 (TRT00001918!), Lo 105.2 (TRT00001917!); Lane Co. Eugene, 1993-05-07, Love 9304 (TRT00002644!), 2003-05-13, Love C2003-12 (TRT00002646!), C2003-13 (TRT00002647!); 2003-06-01, Zika 19571 (TRT00001890!); Linn Co., Cogswell-Foster Preserve (OR1), 1987-04-7, 1987-04-27, 1987-09-20, Love 8707 (TRT00001895!, TRT00001907!, TRT00001912!), 8714 (TRT00001897!, TRT00001899!, TRT00002643!), 8715 (TRT00001901!, TRT00001902!, TRT00001910!), 8716 (TRT00001894!, TRT00001913!), 8717 (TRT00001900!, TRT00001909!), 8718 (TRT00002645!), 8719 (TRT00001893!, TRT00001905!, TRT00001906!), 8720 (TRT00001904!), 1993-05-18, Barbour, Evans & Love 93064 (TRT00001896!), 1997-07-27, Love 9726 (TRT00002196!); 2004-06-10, **Lo, Dickinson & Nguyen 71 (TRT00002650!)**, 73 (TRT00002660!), 76 (TRT00002658!), 77 (TRT00002659!), **79 (TRT00002657!)**, 81 (TRT00002655!), 82 (TRT00002656!), 84 (TRT00002653!), **85 (TRT00002654!)**; 2009-09-03, **Dickinson & Dickinson** 2009-22 (TRT00002555!), 2009-23 (TRT00002556!), 2009-24 (TRT00002557!), 2009-28 (TRT00002560!), 2009-33 (TRT00002565!), 2009-34 (TRT00002566!), **2009-36 (TRT00002568!)**, 2009-38 (TRT00002570!), 2009-39 (TRT00002571!), 2009-41 (TRT00002573!), 2009-42 (TRT00002572!), 2009-43 (TRT00002575!); Marion Co., Salem, 2003-05-01, Zika 18296 (TRT00001889!). **Washington:** Clark Co., 2003-06-01, Zika 18431 (TRT00001891!).
